# Supplementary material for: scRNA-Seq of Cultured Human Amniotic Fluid from Fetuses with Spina Bifida Reveals the Origin and Heterogeneity of the Cellular Content
Source: Cells. 2023 Jun 7;12(12):1577. doi: 10.3390/cells12121577 (PMC10296558; doi:10.3390/cells12121577)
Supplement: Supplementary file 1 [file cells-12-01577-s001.zip › cells-2106972-supplementary/SupplementaryMaterial.pdf]

scRNA-Seq of cultured human amniotic fluid from fetuses with spina bifida reveals the origin and heterogeneity of the cellular content

Athanasia Dasargyri [1], Daymé González Rodríguez [2], Hubert Rehrauer [2], Ernst Reichmann [1], Thomas Biedermann [1,3] and Ueli Moehrlen [1,3,4,5,*]

[1] Tissue Biology Research Unit, Department of Surgery, University Children's Hospital Zurich, 8032 Zurich, Switzerland
[2] Functional Genomics Center Zurich, ETH Zurich and University of Zurich, 8057 Zurich, Switzerland
[3] Faculty of Medicine, University of Zurich, 8006 Zurich, Switzerland
[4] Zurich Center for Fetal Diagnosis and Therapy, University of Zurich, 8006 Zurich, Switzerland
[5] Pediatric Surgery, University Children's Hospital Zurich, 8032 Zurich, Switzerland
* Correspondence: ueli.moehrlen@kispi.uzh.ch; Tel.: +41-44-266-71-11

Corresponding author:

Ueli Moehrlen, MD

Department of Surgery

University Children's Hospital Zurich

Steinwiesstrasse 75

8032 Zurich

Switzerland

phone +41 44 266 71 11

ueli.moehrlen@kispi.uzh.ch

Conflict-of-interest statement:

The authors have declared that no conflict of interest exists.

Supplementary Table S1. Summary of information of the five amniotic fluid samples used for the scRNA-Seq

Sample name	AF volume (ml)	Gestation time (w + d)	Type of neural defect	Sex of the fetus	Time in culture (d)	# of cells detached	# of cells sequenced	# of cells that passed the QC
SBA1	90	24 + 1	MS	f	9	500,000	8237	8129
SBA2	22	24 + 0	MMC	m	10	100,000	8607	8533
SBA3	35	24 + 3	MMC	m	6	100,000	8193	7685
SBA4	50	24 + 6	MS	f	7	200,000	11486	10457
Normal	5	20 + 6	none	f	11	250,000	8993	8883

MS: myeloschisis, MMC: myelomeningocele, f: female, m: male

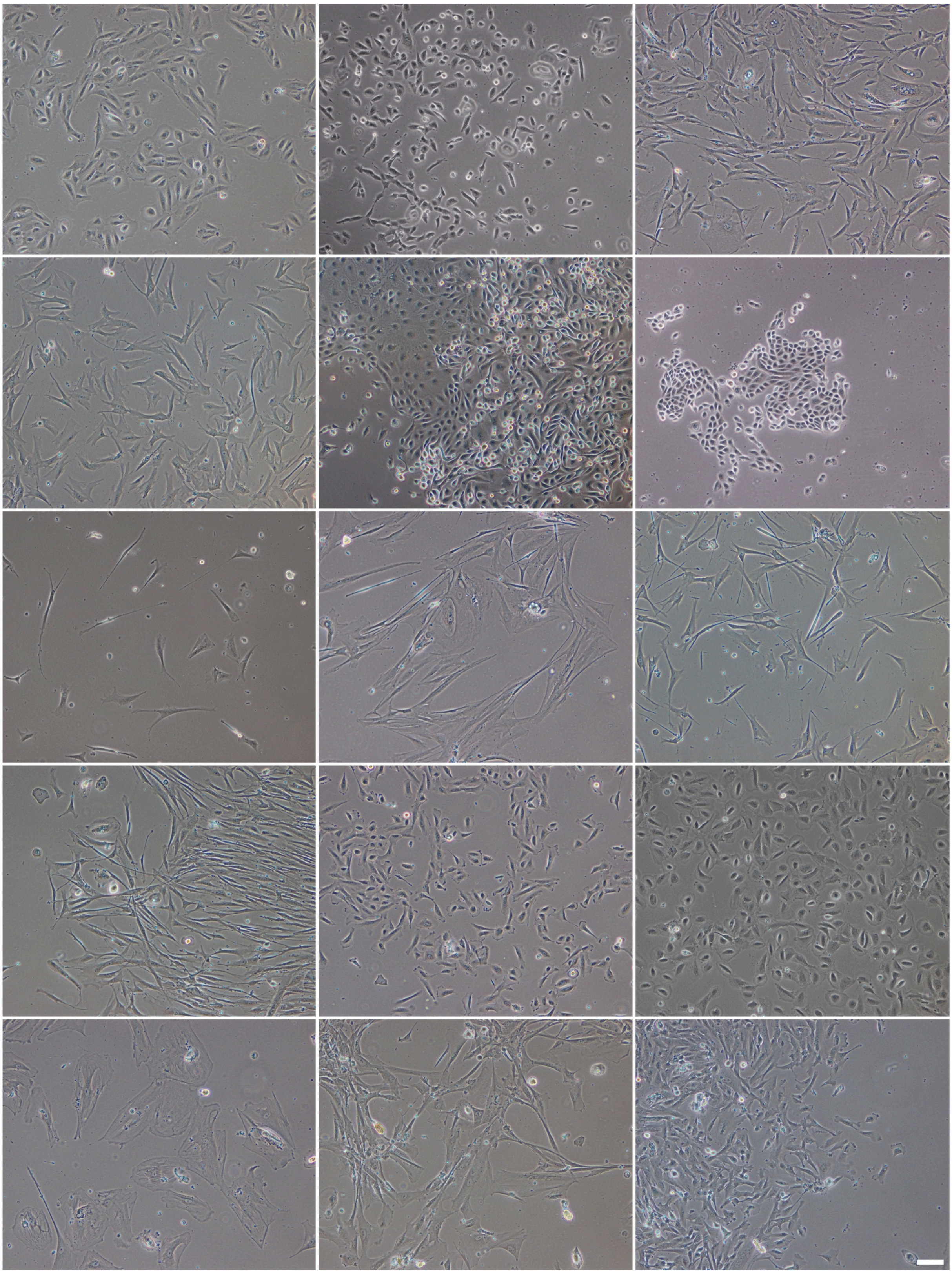

Figure S1. Images of AFC cultures at passage 0 from fetuses with SBA. Fresh AF was centrifuged, plated in Chang C medium and kept in culture until cell colonies appeared. AFC cultures consisted of cell types of variable morphology that grew in tightly packed colonies or as individual cells Scale bar: 100 µm (same in all panels).

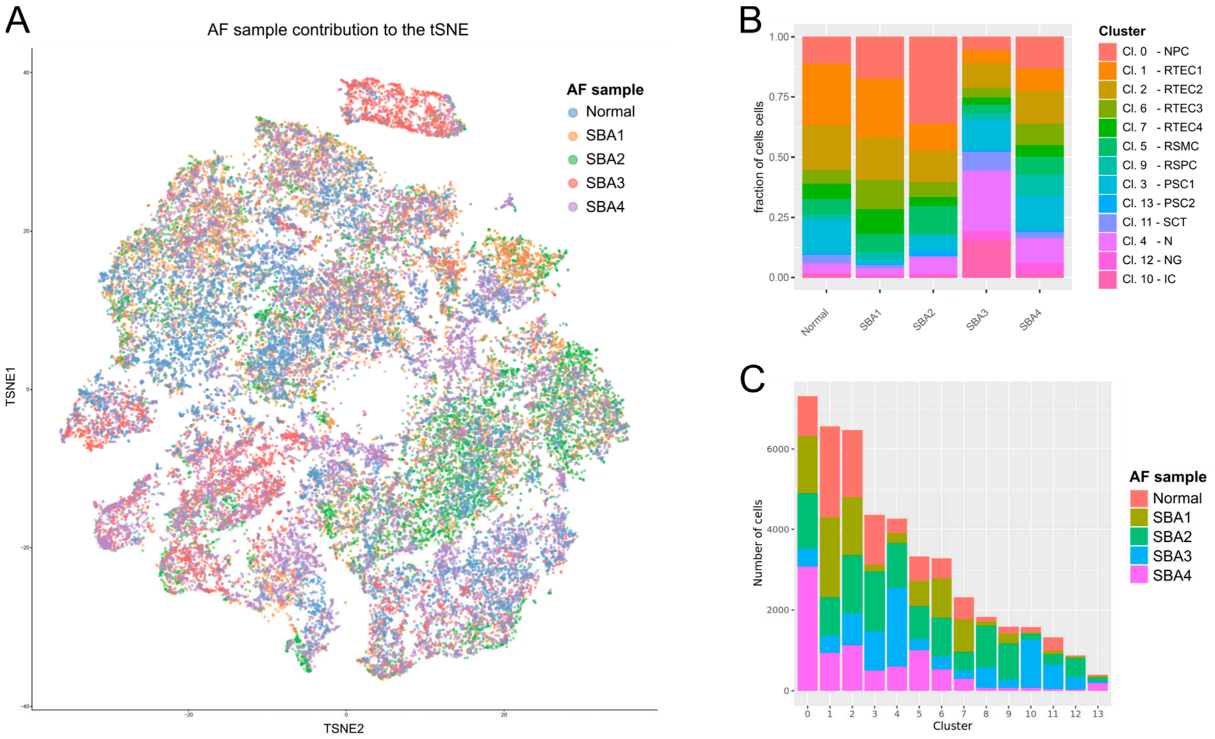

Figure S2. Global analysis of single-cell expression profiles in cultured human AF cells.

(A) t-SNE plot of single cells in the integrated dataset from the five AF samples. Each dot represents a cell and the different colors are assigned to different AF samples. The five samples contribute in a comparable way in populating each region of the graph, showing that the integration was successful.

(B) Stacked bar chart depicting the fraction of cells of all clusters in each AF sample.

(C) Bar chart of the number of cells in each cluster, showing the contribution of each AF sample in the 14 detected clusters (including cluster 8) in the integrated dataset from the five AF samples.

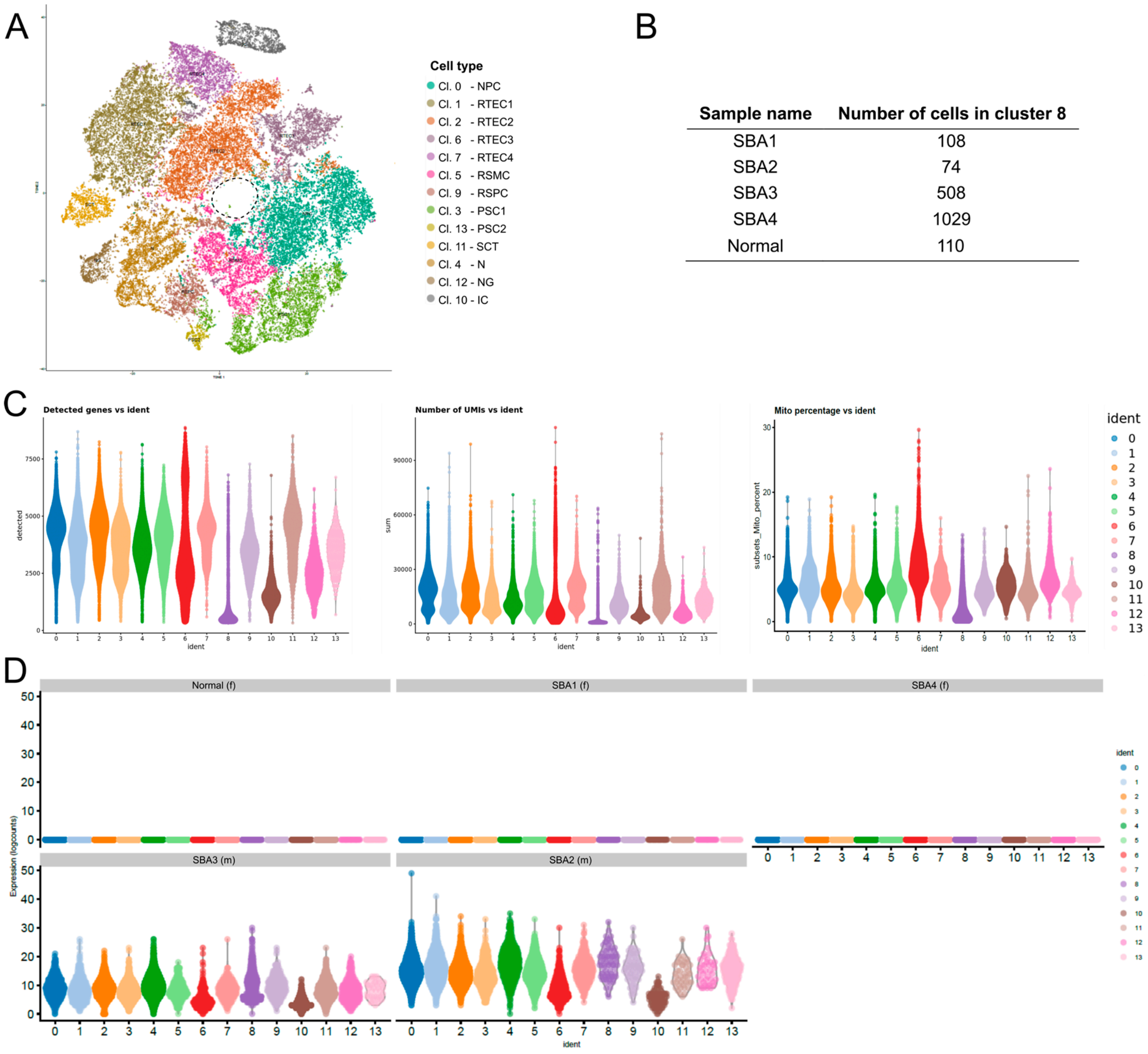

Sample name	Number of cells in cluster 8
SBA1	108
SBA2	74
SBA3	508
SBA4	1029
Normal	110

Figure S3. Quality control of single-cell expression profiles in cultured human AF cells.

(A) t-SNE plot of single cells in the integrated dataset from the five AF samples, as shown in Figure 1. The black dashed line marks cluster 8, which consists of low-quality cells and was removed from the analysis.

(B) Number of cells of each AF sample in cluster 8. The low-quality cells cluster has cells from all samples.

(C) scRNA-seq quality control plots. Violin plots depicting the number of detected genes per cluster (left), the number of UMI per cluster (middle) and the percentage of mitochondrial genes per cluster (right) in the integrated dataset from the five AF samples. Cluster 8 is aberrant in terms of total UMI count, total genes detected, and percentage of mitochondrial genes, confirming that it consists of low-quality cells. Cluster 8 was therefore removed from all subsequent analyses.

(D) Violin plots showing the expression of the male-specific gene *RPS4Y1* per cluster in each of the five AF samples (two from male and three from female fetuses) including cluster 8 (low-quality cells). The gene is expressed in all clusters, indicating that also cells in cluster 8 are of fetal origin.

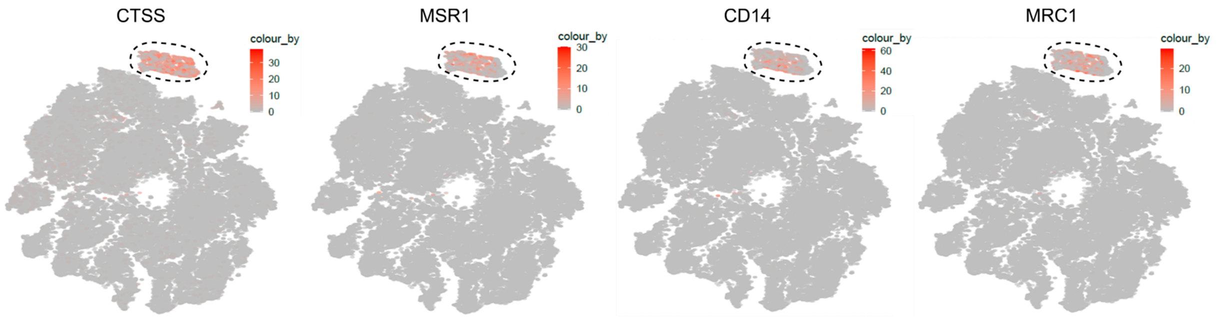

Figure S4. Macrophage markers in cluster 10.

t-SNE plots of all single cells with the expression of representative macrophage-specific marker genes (CTSS, MSR1, CD14, MRC1). Black dashed lines mark the macrophage cluster.

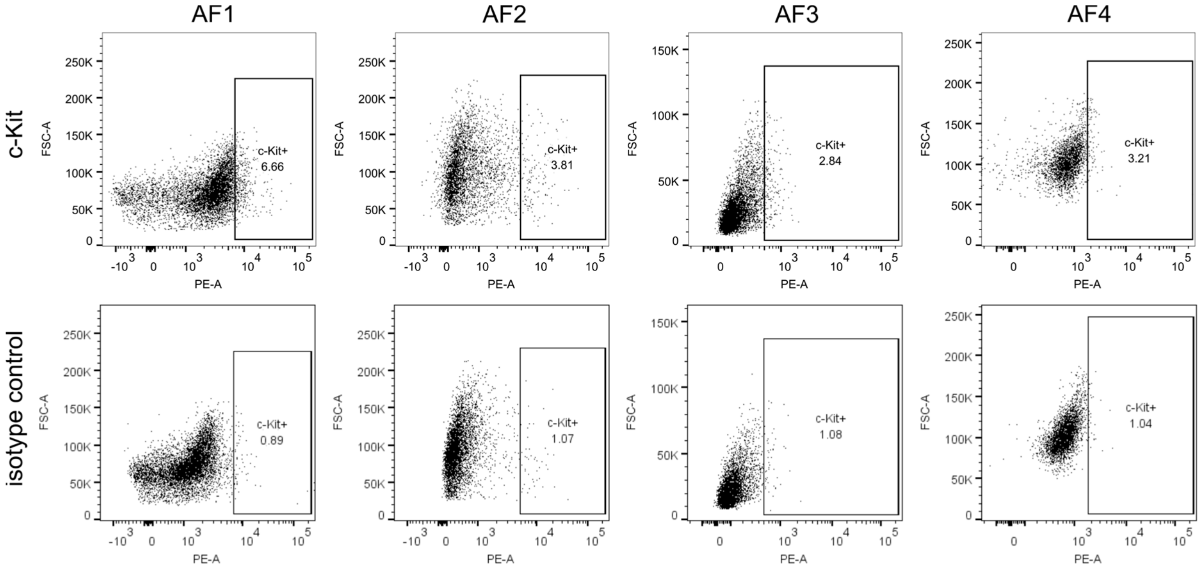

Figure S5. Expression of c-Kit in cultured AF cells at P0 by FACS.

(A) FACS plots showing c-Kit staining in four AF samples from SBA fetuses of gestational age 23 - 26 weeks (different AF samples than those used for scRNA-seq). The first plot is the one shown in Figure 5B of the main text. The gate for c-Kit positive cells was set at 1% using the isotype control antibody. The same gating was used for the cell sorting. The c-Kit antibody has been validated before using human melanocytes (Cell Rep. 2022 Mar 1;38(9):110419).
